# Living Kidney Donor Knowledge of Provided Information and Informed Consent: The PRINCE Study †

**DOI:** 10.3390/jcm11030698

**Published:** 2022-01-28

**Authors:** Emerentia Q. W. Spoon, Kirsten Kortram, Sohal Y. Ismail, Daan Nieboer, Frank C. H. d’Ancona, Maarten H. L. Christiaans, Ruth E. Dam, Hendrik Sijbrand Hofker, Arjan W. J. Hoksbergen, Karlijn Ami van der Pant, Raechel J. Toorop, Jacqueline van de Wetering, Jan N. M. Ijzermans, Frank J. M. F. Dor, on behalf of the Dutch Working Group Informed Consent for Live Donor Nephrectomy (“PRINCE”)

**Affiliations:** 1 Erasmus MC University Medical Centre, Department of Surgery, 3015 GD Rotterdam, The Netherlands; richa_spoon@hotmail.com (E.Q.W.S.); k.kortram@franciscus.nl (K.K.); j.ijzermans@erasmusmc.nl (J.N.M.I.); 2 Erasmus MC University Medical Centre, Department of Psychiatry, 3015 GD Rotterdam, The Netherlands; s.ismail@erasmusmc.nl; 3 Erasmus MC University Medical Centre, Department of Public Health, 3015 GD Rotterdam, The Netherlands; d.nieboer@erasmusmc.nl; 4 Radboud University Medical Centre, Department of Urology, 6525 GA Nijmegen, The Netherlands; f.dancona@hotmail.com; 5 Maastricht University Medical Centre, Department of Internal Medicine, 6229 HX Maastricht, The Netherlands; m.christiaans@mumc.nl; 6 Leiden University Medical Centre, Department of Nephrology, 2333 ZA Leiden, The Netherlands; R.E.Dam@lumc.nl; 7 University Medical Centre Groningen, Department of Surgery, 9713 GZ Groningen, The Netherlands; h.s.hofker@umcg.nl; 8 VU Medical Centre, Department of Surgery, 1081 HV Amsterdam, The Netherlands; a.hoksbergen@vumc.nl; 9 Amsterdam University Medical Centre, Renal Transplant Unit, Department of Internal Medicine, 1105 AZ Amsterdam, The Netherlands; k.a.vanderpant@amc.uva.nl; 10 Amsterdam University Medical Centre, Renal Transplant Unit, Department of Nephrology, 1105 AZ Amsterdam, The Netherlands; 11 Utrecht University Medical Centre, Department of Surgery, 3584 CX Utrecht, The Netherlands; R.J.Toorop@umcutrecht.nl; 12 Erasmus MC University Medical Centre, Department of Nephrology, 3015 GD Rotterdam, The Netherlands; j.vandewetering.1@erasmusmc.nl; 13 Department of Surgery and Cancer, Imperial College, London SW7 2AZ, UK

**Keywords:** education, kidney donation, transplantation, informed consent, living donation, health literacy, long-term risk, live donor nephrectomy

## Abstract

Background: Informed consent for living kidney donation is paramount, as donors are healthy individuals undergoing surgery for the benefit of others. The informed consent process for living kidney donors is heterogenous, and the question concerns how well they are actually informed. Knowledge assessments, before and after donor education, can form the basis for a standardized informed consent procedure for live kidney donation. Methods: In this prospective, a multicenter national cohort study conducted in all eight kidney transplant centers in The Netherlands, we assessed the current status of the informed consent practice for live donor nephrectomy. All of the potential living kidney donors in the participating centers were invited to participate. They completed a pop quiz during their first outpatient appointment (Cohort A). Living kidney donors completed the same pop quiz upon admission for donor nephrectomy (Cohort B). Results: In total, 656 pop quizzes were completed (417 in Cohort A, and 239 in Cohort B). The average donor knowledge score was 7.0/25.0 (±3.9, range 0–18) in Cohort A, and 10.5/25.0 (±2.8, range 0–17.5) in Cohort B. Cohort B scored significantly higher on overall knowledge, preparedness, and the individual item scores (*p* < 0.0001), except for the long-term complications (*p* = 0.91). Conclusions: Donor knowledge generally improves during the live donor workup, but it is still quite disappointing. Long-term complications, especially, deserve more attention during living kidney donor education.

## 1. Introduction

Informed consent is a cornerstone of medical practice, and it is required for surgical interventions [1]. Patients should be correctly informed about the specific details, risks, and alternatives of a procedure, first, in order to be able to make a balanced decision as to whether or not to undergo the procedure, and second, in order to prepare them for the procedure and the postoperative course. Living donors are (generally) healthy individuals, from whom organs are removed for the benefit of others. This warrants an especially vigilant approach to the informed consent process for live donor nephrectomy.

A person providing consent should be “fully informed”, “free of coercion”, and “competent” [2], but there is no consensus on the details to be provided during the process, nor on the manner in which these should be delivered. Informed consent procedures vary per country, per center, and even per individual healthcare professional [3,4]. There are also many different guidelines outlining the matters that should be disclosed to potential donors, but the details are often not specified, and they can vary per guideline [5,6,7,8,9]. A recent study demonstrates that living kidney donors underestimate the complications and risks of a living kidney nephrectomy [10]. Moreover, Surman et al. argue that renal and liver transplant recipients have significant limitations to their knowledge about the postoperative situation [11]. The relative risk of mid- and long-term health risks are increased after live kidney donation [12,13,14,15,16,17]. However, the absolute risk of ESKD is lower than for the general population [18]. However, Muzaale et al. demonstrate a similar increased risk for ESKD compared to well-matched controls [19]. The questions are related to whether or not the necessary information has been provided correctly; whether or not donors understand and remember it; and whether or not they selectively filter information and miss particular risks associated with donation [20,21,22,23].

The importance of the informed consent procedure is based on the Sidaway case (1985). The plaintiff brought action against the hospital and the surgeon because she had not been warned of all of the inherent risks of the procedure [24]. During the operation, a complication with a <1% a priori risk occurred, which left her severely disabled. She demanded compensation for her personal injury because of the surgeon’s failure to disclose all of the surgical risks, and she further strengthened her claim, stating that if the surgeon had informed her of all of the possible risks, she would not have consented to the procedure. Another example, the Montgomery case, concerned a woman with type 1 diabetes who was giving birth, which was complicated by shoulder dystocia, which resulted in her child suffering from brain damage [25]. Because the risk of serious consequences from shoulder dystocia (such as brain damage) was believed to be very small, it was not discussed by the consultant. Thus, because she was not informed of all of the possible complications and treatment options, Mrs. Montgomery was not able to make a fully informed decision. These cases emphasize the importance of discussing the risks of a procedure with patients, and of focusing the process of consent on the individual patient’s concerns [26]. The standardization of the informed consent format will greatly improve the quality of care for living kidney donors [27,28], and should facilitate donor-tailored education. Thus, the format should be a guideline with room for individual adjustments, rather than a strict law.

In our pilot study, the living donor (*n* = 46) knowledge scores were low, regardless of which, or how many, details had been disclosed to them, or which healthcare professional provided them with the information (Kortram et al., unpublished). We conclude that donors may not be sufficiently informed at the time of consent, as is described by others [27,29,30,31,32].

Therefore, the objective of the PRINCE study was to evaluate the informed consent procedure for potential living kidney donors in all Dutch kidney transplant centers, with a focus on the donation procedure, donor knowledge, and donor satisfaction.

The ultimate goal is to improve the informed consent procedure by developing a standardized informed consent procedure. This may, in turn, aid healthcare professionals in delivering tailor-made information to potential donors.

## 2. Methods

Approval for the study was obtained from the Medical Ethics Committee (MEC) of the Erasmus MC University Medical Centre, Rotterdam, The Netherlands (MEC-2014-538). Secondary approval was obtained from the MECs of the other seven participating centers. The study is a prospective multicenter observational cohort study, conducted over 17 months, in all eight kidney transplant centers in The Netherlands (all university medical centers). The detailed study protocol was published previously [33].

The manner of obtaining informed consent in the eight Dutch transplant centers was assessed through interviews with the (para)medical staff in each transplant center, and by observation on site.

This article reports on the two main cohorts (Cohort A and B) of the study (Cohorts 1 and 3 in the protocol) [33]. Cohort 2 could not be completed, as the center participation in this cohort was too low, which was most likely due to the nature of the study (recorded informed consent conversations). For the figure depicting the study design, we refer the reader to our published study protocol [33].

Cohort A consisted of potential living kidney donors prior to their first outpatient visit, and Cohort B entailed a group of donors on the day of admission for donor nephrectomy. Minimum sample sizes were calculated in order to provide an adequate reflection of the general population: Cohort A consisted of 50 donors per center, and, thus, 400 donors, and Cohort B consisted of a set number of donors per center, which was based on each center’s volume in the preceding year, and which resulted in a minimum of 200 donors (details described in the protocol [33]). Because of the duration of the inclusion period, some of the donors of Cohort A also eventually ended up being included in Cohort B.

After obtaining their informed consent for participation in the study, the potential live kidney donors were asked to complete a baseline questionnaire and a pop quiz. The baseline questionnaire consisted of questions regarding gender, the relationship to the recipient [34], education, employment, religion, household constitution, and charity activities. The pop quiz consisted of five open-ended questions regarding live kidney donation, which concerned the surgical technique, the complications (including short-term and long-term), the hospital stay, and the duration of convalescence. Every answer to the questions regarding the abovementioned topics was given an equal weighing factor. In addition, the donors were asked to indicate how well prepared they felt for the donation procedure by means of a visual analogue scale (VAS). A scoring system was developed to calculate the pop quiz, which was based on the pilot study. A maximum of five points were awarded for each of the five sections. All of the Cohort B donors received an evaluation and satisfaction questionnaire, containing open questions, three months postoperatively. If no response was obtained, a reminder was sent after another three months.

The primary outcome of this study was the donor knowledge, which was measured by the pop-quiz scores. The secondary outcomes were the manner of obtaining consent for donor nephrectomy in the different transplant centers, and the donor satisfaction with this procedure.

### Statistical Analysis

The statistical analysis was performed using SPSS, version 21, and R, version 3.1.2. The dichotomous data and counts are presented in frequencies. The continuous data are presented in means and standard deviations (SDs), or median values and ranges.

The differences between the scores and the preparation values were compared by the independent sample *t*-test, the pairwise comparison *t*-test, or the one-way ANOVA. Chi-squared tests were conducted to compare the differences in the frequencies of the individual complications between Cohort A and Cohort B. To correct for overlapping donors, linear mixed effect models were used to account for the correlations between the patients present in both cohorts. Missing values were imputed using single imputation. Additional McNemar tests were performed for the overlapping donors in both cohorts in order to compare the individually mentioned frequencies at the different time intervals. The McNemar test compares the number of those who first scored positive (i.e., who mentioned that specific complication), and then scored negative (i.e., did not mention that specific complication), with the number who first scored negative, and then scored positive. A significant increase or decrease was concluded if *p* < 0.05. A multivariate analysis was performed to assess whether the donor scores were influenced by specific characteristics. Linear regression was used. Every factor with a univariate *p*-value < 0.1 was included in the multivariate model. To investigate the potential differences between centers, the center-specific estimates of the multivariable model were assessed.

## 3. Results

### 3.1. Informed Consent Procedure per Center

Table 1 provides an overview of the local protocols for each center. The local situation in the eight participating centers varies with regard to the donor nephrectomy itself, but also with regard to the specific setup of the informed consent procedure for live kidney donation.

### 3.2. Donors

A total of 656 pop quizzes were completed. A total of 417 living kidney donors were included in Cohort A, and 239 were included in Cohort B. Forty donors from Cohort A also progressed to Cohort B, and therefore the data of these two different timepoints can be compared for these 40 donors.

Table 2 provides an overview of the baseline characteristics of each cohort.

### 3.3. Preparation for the Donation Procedure

Understandably, the donors in Cohort A did not feel very well prepared: 5.6/10 (±2.5). The donors in Cohort B, having received all possible education, did report a significantly better feeling of preparedness: 8.2/10 (±1.3) (*p* < 0.0001).

### 3.4. Pop-Quiz Scores

Table 3 presents an overview of the scores per cohort, and per subdivision.

#### 3.4.1. Cohort A

The mean overall knowledge score for the Cohort A donors, prior to the first visit at the nephrology outpatient clinic, is 7.0/25 (±3.9, range 0–18). The donors scored best on “convalescence”, and worst on “long-term complications” (Table 3).

In terms of the short-term complications, fatigue was mentioned most by the donors in Cohort A (*n* = 141, 34%), followed by pain (*n* = 80, 19%), and infections (*n* = 70, 17%). The risk of death was only mentioned by 21 donors (5%). The frequencies of all the complications mentioned are displayed in Table 4. Aside from those complications included in the scoring system, the donors mentioned additional problems that they thought might occur.

Long-term complications: The eventual risk for developing ESKD was described by 66 Cohort A donors (16%). Other long-term complications were only incidentally mentioned (Table 4).

#### 3.4.2. Cohort B

The mean overall donor knowledge score in Cohort B, on the day of admission for donor nephrectomy, is 10.5/25 (±2.8, range 0–17.5). They scored best on “duration of admission”, and again, worst on “long-term complications” (Table 3).

With regard to the short-term complications, the order of the most frequently mentioned complications was more or less comparable with Cohort A, but each complication was mentioned more often than in Cohort A: fatigue (*n* = 103, 43%), bleeding (*n* = 74, 31%), and pain (*n* = 72, 30%) were the top three. The risk of mortality was mentioned by 32 donors (13%) (Table 4). Nausea was, again, the most frequently mentioned additional complication.

With regard to the long-term complications, the risk of renal failure was the most frequently mentioned; however, it was mentioned slightly less often than at the outpatient clinic: *n* = 35, 15%. The other complications were, again, only mentioned sporadically (Table 4).

The average overall Cohort B knowledge score was significantly higher than the average Cohort A score (*p* < 0.0001, Table 3). This was also true for each of the individual item scores, with the exception of “long-term complications”: the average score was 0.2 for both the outpatient and the admission cohorts (Table 3).

#### 3.4.3. Overlapping Donors

The donors who were included in Cohort A and Cohort B (*n* = 40) scored, overall, significantly better the second time they completed the pop quiz, on the day of the admission for the donor nephrectomy: 11.0 (±2.9) versus 8.7 (±3.1), *p* = 0.001. However, seven donors actually scored worse on admission than at the outpatient clinic, with a mean difference of 3.68 (±1.87, range 0.75–6). This decrease was seen in all the individual item scores. The risk of bleeding was the only individual item that was mentioned significantly more often upon admission than prior to the first outpatient consult: 40% versus 15% of donors recalled this risk on the Cohort B pop quiz. Table 5 provides an overview of all the individual complications, and their mentioning frequencies for the longitudinal cohort.

### 3.5. Score Correlation

The baseline scores (Cohort A) varied per center. The scores ranged from 6.4 (±3.1) in the lowest scoring center, to 8.5 (±2.2) in the highest scoring center (overall, *p* = 0.02). The center volume (i.e., the number of donor nephrectomies performed per year) did not influence the donor score. Younger donors scored better than older donors (*p* = 0.02), as did donors with higher educational levels (defined as “University of applied sciences or University”) (8.1 (±3.3) versus 6.4 (±4.0) (*p* < 0.0001)), and donors who were employed (7.3 (±3.8) versus 5.4 (±3.9) for unemployed donors, and 6.0 (±4.0) for retired donors, *p* = 0.001). However, after the multivariable analysis, only a younger age, a higher educational level, and registration as a confirmed (deceased) organ donor were associated with higher pop-quiz scores. Table 6 presents the results of the multivariate analysis for both cohorts.

In Cohort B, registered organ donors (10.8 (±2.8) versus unregistered organ donors, 9.9 (±2.7), *p* = 0.008), employed donors (10.9 (±2.5) versus 9.0 (±3.4) for unemployed donors, and 9.5 (±2.8) for retired donors, *p* = 0.001), and donors living with children under 18 (11.3 (±2.5) versus 10.2 (±2.8), *p* = 0.009) scored significantly higher in the univariate analysis than donors without these characteristics. After the multivariate analysis, current employment, a household with children under 18, and registration as a confirmed (deceased) donor were related to higher pop-quiz scores. Differences per center were not observed in this cohort (Table 6).

### 3.6. Descriptive Results

On the basis of the feedback on the question regarding the surgical technique in our pilot study (Kortram, unpublished), we rephrased the question for the PRINCE study; only two donors stated that the technique had been explained to them, but they did not elaborate on what this explanation entailed.

In Cohort A, 28 (7.5%) donors did not know the answer to (some of) the questions about convalescence, and 115 (31.2%) did not know about the long-term complications. In Cohort B, all of the donors provided an answer to the question about admission; only two (0.9%) had no idea about convalescence, four (1.8%) had no idea about the surgical technique, 14 (6.4%) had no idea about the short-term complications, and 44 (20%) had no idea about the long-term complications.

One donor in Cohort B answered the surgical technique question with, “*this is not important to me at all*”, and another donor answered something similar to the answer for the “risks and complications” question: “*I do not think about this, it is not important to me. I have been told about this, but risks and complications are minimal*”. In Cohort A, one donor said: “*I do not really care about long term consequences; if they occur, we will see what we can do about it then*”. These answers, although given by the vast minority, suggest that perhaps some donors do not want to know all the specific details, and that they may still feel informed and prepared.

### 3.7. Evaluation and Satisfaction

The evaluation questionnaires were returned by 158 Cohort B donors (72%). Overall, the average satisfaction with the informed consent procedure was 8.1/10 (SD 1.6, range from 0.6–10). Although the majority of donors were positive, some raised valid concerns.

One donor in the kidney paired exchange program underlined the importance of standardization, stating that the “information provision was inadequate. It would be a suggestion to create a checklist with items that have and have not been discussed, and items that should still be addressed”. Some donors who developed postoperative complications claimed that these were not disclosed to them during the informed consent process: “it was repeatedly stated that no complications were expected, but two out of three complications [that occurred] commonly known prior to surgery. Information was too optimistic, and not very realistic”. However, donors who had not experienced complications also indicated that they had wished to hear more about potential complications prior to the donation procedure. On the other hand, some donors indicated that they had received too much information: “I received so much information that a possible shortage in knowledge is due to the amount of information”.

Some donors addressed the fact that not enough attention had been paid to the convalescence period. Another recurring statement was that, although donors remembered being told about certain risks or complications, they had assumed that these would not occur: “*I was stubborn and did not believe that the provided information would apply to me*”, or “*you always know that there can be complications, but you never think it will happen to you*”.

## 4. Discussion

The PRINCE study is, to the best of our knowledge, the first, and the largest, available prospective nationwide cohort study testing living kidney donors on their knowledge regarding the surgical procedure, the postoperative course, the possible complications, and the long-term results, with special attention paid to informed consent. It is also the first study using open questions, which prompted the donors to describe the answers in their own words.

All of the potential donors were educated on the basis of the center-specific informed consent procedures, although the national guidelines were always followed. The baseline knowledge scores varied between centers, possibly because of the fact that some donors had received information in the referring hospital, rather than in the transplant center. The time between the first visit to the outpatient clinic and the actual donation was expected to be quite long in at least a part of the population. This is because potential donors are often screened well before the donation takes place. Therefore, we chose to include all of the potential living kidney donors who presented for the first time at the outpatient clinic (Cohort A), and all of the donors who had already successfully completed the donation procedure (Cohort B). A longitudinal inclusion would, of course, have been desirable, but it was not technically feasible because the inclusion period would have taken years to complete.

Our study demonstrates that the donor knowledge significantly improves during the preoperative workup; overall, the scores were significantly higher on admission than at the nephrology outpatient clinic, prior to the receipt of any information. However, large knowledge deficits are still present. Previous studies have also demonstrated substantial gaps in donor knowledge [10,35,36], but it has also been argued that their decision-making strategies differ from regular patients [21,22,37]. The question is whether or not the substantial knowledge gap of living kidney donors is caused by insufficient information provided by healthcare professionals, by donors not understanding or remembering relevant details, or by selective perception (patients only hear what they wish to hear in order to confirm their decision to donate). In our view, it is important to understand how the informed consent process might break down this barrier and facilitate truly informed decision making so that we can limit the variation in donor literacy. Unfortunately, to the best of our knowledge, there is no literature on how to overcome this type of learning bias. Even if decision-making strategies may differ between donors and “regular” patients, knowledge deficits are also encountered in other patient categories. Lee et al. published a similar study on breast cancer patients prior to undergoing mastectomies, who received a validated test to assess their knowledge about breast reconstruction. The overall knowledge score was 58.5% (compared to 41.6% in Cohort B of this study), but the score for the risk of complications was only 14.3% (compared to 22% in this study) for the short-term complications, and 4.8% for the long-term complications [38]. Therefore, knowledge differences are apparent between specified and unspecified donors (Table 6). A likely explanation is that unspecified donors do not have an emotional relationship with the recipient, so they make the choice to donate regardless of whether a recipient needs the kidney quickly. In addition, they all undergo a mandatory evaluation by a psychologist or psychiatrist.

The large multicenter cohort and the prospective nature of this study are definite strengths. The Netherlands is a leading country when it comes to live kidney donations (33 per million of the population [39]), and this study, which includes all of the Dutch kidney transplant centers, provides a reliable overview of the current national situation.

We acknowledge a number of limitations. First, nonvalidated questionnaires were used. A validated knowledge test is available for living kidney donors [10]; however, the PRINCE study wished to receive more specific information about the informed consent, rather than knowledge about, e.g., ESKD, and we piloted the study in one transplant center. The validation of a knowledge test with open questions is virtually impossible because donors may learn or forget information at different timepoints, and because their knowledge is (partly) dependent on the information they have received from their transplant team. A multiple-choice scoring system would have made it easier to compare the donor knowledge at different timepoints. However, with open-ended questions, we collected more reliable information about the actual gained knowledge, rather than recollections of the terms that were used during the preoperative workup.

The second limitation arises from the fact that potential donors could misinterpret open questions. For example, some donors left some of our questions open, or only wrote down one or two complications. Despite this, overall, we are convinced that our open-ended questionnaire provided us with more valid information about donor knowledge than a multiple-choice questionnaire because the patients could then easily recognize the predefined answers, instead of actually remembering the provided information. The risk of death, for instance, was only mentioned in 48 pop quizzes (8%), compared to 18% in the pilot study. A possible explanation is that donors may choose to ignore this risk. Even though they might actually be aware of it, writing it down makes it “real”. Moreover, donors may not regard death as a realistic concern: they are more focused on dealing with pain, fatigue, recovering, and getting back to work.

The donor satisfaction with the informed consent procedure was, with an average score of 8.1/10, quite high, even though the donor knowledge was lower than expected. This has been demonstrated before by Amir et al.: only 40.5% of patients understood the provided information, but 93.5% were satisfied with the informed consent process [40]. However, even if a donor is satisfied and does not wish to receive more information about the procedure, we still have an obligation to provide all of the necessary information, especially since some donors retrospectively report that they wish they had had more information preoperatively [36].

In our pop quiz, all of the answers to our five main topics were given equal weighing factors for the final scoring. Because of this, all of the possible complications were assigned the same importance. We thus obtained a good estimate of the number of remembered complications, instead of a different calibration of the complications into “significant” and “not so frequent” complications. However, the assessment of importance would have been based on the opinions of healthcare professionals, rather than on the opinions of donors. By using our approach, the PRINCE study provides important information about which complications matter most to living kidney donors at different times. We detected key deficiencies in the informed consent process, which can feed into the redesign of the educational process for living kidney donors.

One of the main issues with a standardized format is that no two donors are alike, and neither are their information needs and wishes. A recent study assessing the information needs in cancer patients prior to surgery demonstrates that the patients were not so much interested in the technical details and the short-term morbidity but were more interested in the survival data and the long-term quality of life [41]. Oncological patients may not be comparable to living kidney donors because they need the procedure to survive or to extend their lives. They may see some risks as inevitable, and they may not see any added value in knowing about them. Living kidney donors do not need this operation, and they have often already decided to help others before receiving any information.

The PRINCE study provides a basis for improving living donor education and the informed consent process. Providing additional information, prior to the surgical consult, may be a possible solution. In a study on cardiac surgery patients, those patients receiving extended written information were, overall, more satisfied with the informational process [42]. However, this study did not evaluate their knowledge about the surgery. In some centers, donors are asked for informed consent prior to screening (Table 1). This often relates to informed consent for the screening procedure and includes a final consent for the actual donation. Since the informed consent procedure varies widely, there is room for improvement. An educational tool that tests the donor’s knowledge about the donation procedure and the postoperative period may be another key step in improving the informed consent process [43]. The transplant surgeon can then focus on those aspects for which the donor’s knowledge is insufficient. This way, the information provision will still be standardized, but the surgical consult will be donor-tailored, leading to better informed, and likely more satisfied, donors.

One of the next steps could be to evaluate the available guidelines [7,8,9], and update these where appropriate. For instance, the BTS guideline provides a clear overview of the literature on perioperative mortality and morbidity, but it presents just overall percentages of the major complications [7]. The KDIGO guidelines provide information on the long-term risks, but they are less specific on the perioperative complications [9]. It would be helpful to also include complications such as pain and fatigue, which appear to be important to donors. Our recent systematic review and meta-analysis [44] presents an intraoperative complication rate of 2.3%, and a postoperative complication rate of 7.3%. Including more specified data on the perioperative morbidity and the donors’ wishes in these guidelines would further aid transplant professionals in preoperative donor education. In addition, more attention should be given to the long-term consequences of living kidney donation.

## Figures and Tables

**Table 1:** Differences in techniques, information provision, and informed consent procedures per center.

	Employed Techniques	Set-Up Preoperative Visits and Information	Informed Consent How/When/by Whom
Center 1	LD, Mini-open	Consult with TC. If approved: consult with nephrologist and surgeon on the same day (2–8 weeks prior to procedure).	Signed Prior to screening TC
Center 2	HAL	Consult with TC, nephrologist. If there is a wish to continue: discussion in multidisciplinary meeting with surgeon. If approved: last information from surgeon at the clinic (1–2 weeks prior to surgery), or on day of admission.	Signed Prior to screening TC
Center 3	LD, Mini-open	Consult with SN (if unspecified donor: also consult with psychiatrist). Screening tests and consult with social worker. If approved: joint clinic consult with nephrologist and surgeon.	Signed After screening and all consults TC
Center 4	LD, HARP, HAL, Robot	Consult with nephrologist, then consult with TC. If approved: consult with surgeon at outpatient clinic. On day of admission: last information from surgeon and SN on the ward.	Signed Prior to surgical consult Nephrologist
Center 5	HARP	Consult with SN, then consult with nephrologist. Two weeks prior to surgery consult with surgeon.	Signed Prior to surgical consult Nephrologist (Surgeon documents informed consent in EPF)
Center 6	HAL, HARP	First consult with TC. If donor wishes to continue: two-day program, with screening tests and consult with social worker, then nephrologist, SN, and surgeon, in random order. One month prior to surgery consult with TC. Last information from surgeon and TC on day of admission.	Signed Prior to screening TC
Center 7	LD, HAL, HARP	First visit with TC, then consult with nephrologist, then with surgeon.	Signed Prior to surgical consult Nephrologist
Center 8	Mini-open	Work up by SN, approved by nephrologist, 6–4 months prior to surgery consult with surgeon	Explicitly asked SN (Surgeon documents informed consent in EPF)

LD: laparoscopic donor nephrectomy; TC: transplant coordinator; HAL: hand-assisted laparoscopic; HARP: hand-assisted retroperitoneoscopic; SN: specialized nurse; EPF: electronic patient file.

**Table 2:** Baseline characteristics of all (potential) living kidney donors included in this study, specified for the two cohorts (percentages between brackets, unless otherwise defined) ^a^.

	Cohort A *n* = 416	Cohort B *n* = 239	*p*-Value
Gender			
Male	173 (41.6)	110 (46.0)	0.29
Female	243 (58.4)	129 (54.0)	
Age (mean, SD)	53.5 (12.4)	54.1 (11.9)	0.57
Type of donation [34]			
Unspecified	63 (15.1)	50 (20.9)	0.07
Specified	349 (83.9)	188 (78.7)	
Unknown	5 (1.2)	1 (0.4)	
Educational level ^b^			
Lower	284 (68.3)	173 (72.4)	0.33
Higher	130 (31.3)	66 (27.6)	
Current employment			
Yes	291 (70.0)	15 (6.3)	
No	35 (8.4)	171 (71.5)	0.60
Retired	88 (21.2)	53 (22.2)	
Income			
Below average	93 (22.4)	46 (19.2)	
Average	237 (57.0)	138 (57.7)	0.54
Above average	65 (15.6)	43 (18.0)	
Religion			
None	192 (46.2)	115 (48.1)	
Catholicism	93 (22.4)	61 (25.5)	
Protestantism	77 (18.5)	37 (15.5)	
Islam	19 (4.6)	8 (3.3)	0.47
Buddhism	1 (0.2)	3 (1.3)	
Hinduism	5 (1.2)	3 (1.3)	
Other	25 (6.0)	10 (4.2)	
Household constitution			
Alone	85 (20.4)	42 (17.6)	
With children <18	248 (59.6)	146 (61.1)	0.65
Without children <18	81 (19.5)	50 (20.9)	
Registered as deceased organ donor			
Yes	167 (40.1)	96 (40.2)	1.0
No	247 (59.4)	141 (59.0)	

^a^ Not every donor completed every question, and the total numbers may not add up to 413/239 for each item. ^b^ Lower: no education, or primary school, high school, or secondary vocational education; Higher: university of applied sciences or university.

**Table 3:** Pop-quiz scores and item scores for donors included in the two cohorts (mean, SD). The maximum overall score is 25 points, 5 points for each score item.

	Cohort A *n* = 417	Cohort B *n* = 239	*p*-Value
Overall Score	7.0 (3.9)	10.5 (2.8)	<0.0001
Convalescence ^a^	2.9 (1.6)	3.4 (1.3)	<0.0001
Admission ^a^	2.6 (1.7)	3.6 (0.9)	<0.0001
Surgical technique ^a^	0.7 (1.0)	2.2 (1.2)	<0.0001
Short-term complications ^a^	0.7 (0.8)	1.0 (0.9)	<0.0001
Long-term complications ^a^	0.2 (0.4)	0.2 (0.4)	0.91

^a^ For the item scores, the 40 overlapping donors were excluded from the analysis, and the *p*-values were calculated using the independent samples *t*-test. The subscores are thus calculated for 373, versus 199, donors.

**Table 4:** Frequencies of the individual complications mentioned, per cohort. Percentages between brackets.

	Cohort A *n* = 417	Cohort B *n* = 239	*p*-Value
Short-term complications			
Fatigue	141 (33.8)	103 (43.1)	0.2
Pain	80 (19.2)	72 (30.1)	0.02
Infection (NOS)	70 (16.8)	54 (22.6)	0.3
Wound infection	66 (15.8)	71 (29.7)	0.001
Bleeding	51 (12.2)	74 (31.0)	0.03
Thrombosis	39 (9.4)	33 (13.8)	0.08
Pneumonia	36 (8.6)	35 (14.6)	0.23
Urinary tract infection	26 (6.2)	35 (14.6)	<0.0001
Death	21 (5.0)	32 (13.4)	0.57
Damage to other organs	3 (0.7)	1 (0.4)	0.93
Neuropathy/neurapraxia	3 (0.7)	6 (2.5)	0.11
Cardiovascular complications	2 (0.5)	2 (0.8)	0.66
Testicular complaints ^a,b^	0	3 (1.3)	0.8
Long-term complications			
ESKD	66 (15.8)	35 (14.6)	0.87
Chronic pain	11 (2.6)	6 (2.5)	0.74
Hypertension	11 (2.6)	13 (5.4)	0.13
Incisional hernia	4 (1.0)	4 (1.7)	<0.0001
Medication (NSAIDs, AB)	1 (0.2)	3 (1.3)	0.18

NOS: not otherwise specified; ESKD: end-stage kidney disease; NSAIDs: nonsteroidal anti-inflammatory drugs: AB: antibiotics; NA: not applicable. ^a^ Since testicular complaints are a relevant complication only in male donors, the relevant percentage is 2.7% (3 out of 110 males in Cohort B), instead of 1.3% for the whole group of 226 donors. ^b^ Because none of the Cohort A donors reported this complication, a *p*-value could not be computed using the generalized linear model.

**Table 5:** Frequencies of the individual complications mentioned, per cohort, for the 40 overlapping donors. Percentages between brackets.

	Cohort A	Cohort B	*p*-Value
Short-term complications			
Fatigue	18 (45)	22 (55)	0.42
Infection (NOS)	12 (30)	10 (25)	0.79
Pain	12 (30)	11 (27.5)	1
Wound infection	8 (20)	15 (37.5)	0.09
Thrombosis	6 (15)	10 (25)	0.22
Bleeding	6 (15)	16 (40)	0.006
Death	5 (12.5)	6 (15)	1
Pneumonia	5 (12.5)	7 (17.5)	0.69
Urinary tract infection	3 (7.5)	9 (22.5)	0.07
Cardiovascular complications	0	1 (2.5)	1
Neuropathy/neurapraxia	0	3 (7.5)	0.25
Long-term complications			
ESKD	6 (15)	11 (27.5)	0.23
Medication (NSAIDs, AB)	1 (2.5)	0	1
Chronic pain	1 (2.5)	1 (2.5)	1
Hypertension	0	3 (7.5)	0.25

NOS: not otherwise specified; ESKD: end-stage kidney disease.

**Table 6:** Multivariate analysis for total scores, in relation to demographic characteristics, split for the outpatient (Cohort A) and admission (Cohort B) cohorts.

	Univariate	Multivariate Beta	Multivariate
	*p*-Value	(95% CI)	*p*-Value
**Cohort A (*n* = 417)**			
Factor			
Gender			
Age		−20.033	
Specified donation ^a^			
Higher educational level ^b^		1.205	
Current employment ^c^	0.22	0.514	
Income	0.02		0.05
Religion	0.29		
Household	<0.0001		0.002
Registered Donor	0.001	1.724	0.24
Centre	0.26		
1	0.88	ref	
2	0.89	0.507	
3	<0.0001	−0.194	<0.0001
4	0.02	−0.422	0.13
5		0.721	
6		0.306	
7		1.373	
8		1.701	
**Cohort B (*n* = 226)**			
Factor			
Gender	0.44		
Age	0.06	−0.004	0.81
Specified donation ^a^	0.08	−0.812	0.07
Higher educational level ^b^	0.19		
Current employment ^c^	0.001	1.132	0.005
Income	0.93		
Religion	0.29		
Household with children <18 ^d^	0.009	0.944	0.04
Registered Donor	0.008	0.777	0.04
Centre	0.372		

^a^ Compared to anonymous/unspecified donors. ^b^ Compared to lower educational level. ^c^ Compared to unemployed donors. ^d^ Compared to donors living without children <18.

## Data Availability

The data presented in this study are available on request from the corresponding author. The data are not publicly available due to privacy regulations.

## Supplementary Materials

The following supporting information can be downloaded at: https://www.mdpi.com/article/10.3390/jcm11030698/s1. A list with all Collaborators of the Dutch Working Group Informed Consent for Live Donor Nephrectomy (“PRINCE”) is provided in the Supplementary Materials.
