# Supplementary material for: Living Kidney Donor Knowledge of Provided Information and Informed Consent: The PRINCE Study
Source: J Clin Med. 2022 Jan 28;11(3):698. doi: 10.3390/jcm11030698 (PMC8837079; doi:10.3390/jcm11030698)
Supplement: Supplementary file 1 [file jcm-11-00698-s001.zip › jcm-1528422-supplementary.pdf]

Supplementary Materials

Members of the Dutch Working Group Informed Consent for Live Donor Nephrectomy (including authors)

- Emerentia Q. W. Spoon, Department of Surgery, Erasmus MC University Medical Centre, 3015 GD Rotterdam, The Netherlands
- Kirsten Kortram, Department of Surgery, Erasmus MC University Medical Centre, 3015 GD Rotterdam, The Netherlands
- Maarten H. L. Christiaans, Department of Internal Medicine, Maastricht University Medical Centre, 6229 HX, Maastricht, The Netherlands
- Frank C. H. d'Ancona, Department of Urology, Radboud University Medical Centre, 6525 GA, Nijmegen, The Netherlands
- Ruth E. Dam, Department of Nephrology, Leiden University Medical Centre, 2333 ZA, Leiden, The Netherlands
- H John A Dackus, Department of Internal Medicine, Maastricht University Medical Centre, 6229 HX, Maastricht, The Netherlands
- Jeroen Dubbeld, Department of Nephrology, Leiden University Medical Centre, 2333 ZA, Leiden, The Netherlands
- Hendrik Sijbrand Hofker, Department of Surgery, University Medical Centre Groningen, 9713 GZ, Groningen The Netherlands
- Arjan W. J. Hoksbergen, Department of Surgery, VU Medical Centre, 1081 HV, Amsterdam, The Netherlands
- Jaap J Homan van der Heide, Department of Internal Medicine, Department of Nephrology, Renal Transplant Unit, Amsterdam University Medical Centre, 1105 AZ, Amsterdam, The Netherlands
- LW Ernest van Heurn, Department of Surgery, VU Medical Centre, 1081 HV, Amsterdam, The Netherlands; Department of Surgery, Academic Medical Centre Amsterdam, 1105 AZ, Amsterdam, The Netherlands
- Mirza M Idu, Department of Surgery, Academic Medical Centre Amsterdam, 1105 AZ, Amsterdam, The Netherlands
- Jan N. M. Ijzermans, Department of Surgery, Erasmus MC University Medical Centre, 3015 GD, Rotterdam, The Netherlands
- Sohal Y. Ismail, Department of Psychiatry, Erasmus MC University Medical Centre, 3015 GD, Rotterdam, The Netherlands
- Tessa de Jong, Department of Internal Medicine, Department of Nephrology, Renal Transplant Unit, Amsterdam University Medical Centre, 1105 AZ, Amsterdam, The Netherlands
- Caspar WN Looman, Department of Public Health, Erasmus MC University Medical Centre, 3015 GD, Rotterdam, The Netherlands
- J Hillian Nederhoed, Department of Surgery, VU Medical Centre, 1081 HV, Amsterdam, The Netherlands
- Daan Nieboer, Department of Public Health, Erasmus MC University Medical Centre, 3015 GD, Rotterdam, The Netherlands
- S Azam Nurmohamed, Department of Nephrology, VU Medical Centre, 1081 HV, Amsterdam, The Netherlands
- Karlijn Ami van der Pant, Department of Internal Medicine, Department of Nephrology, Renal Transplant Unit, Amsterdam University Medical Centre, 1105 AZ, Amsterdam, The Netherlands
- Desiree Pilzecker, Department of Nephrology, Radboud University Medical Centre, 6525 GA, Nijmegen, The Netherlands

- Juliette D Rabbeljee, Department of Surgery, University Medical Centre Groningen, 9713 GZ, Groningen The Netherlands
- Franka E van Reekum, Department of Nephrology, Utrecht University Medical Centre, 3584 CX, Utrecht, The Netherlands
- Jan Ringers, Department of Nephrology, Leiden University Medical Centre, 2333 ZA, Leiden, The Netherlands
- Geert WH Schurink, Department of Surgery, Maastricht University Medical Centre, 6229 HX, Maastricht, The Netherlands
- Raechel J. Toorop, Department of Surgery, Utrecht University Medical Centre, 3584 CX, Utrecht, The Netherlands
- Philip TR Ulrichts, Department of Internal Medicine, Maastricht University Medical Centre, 6229 HX, Maastricht, The Netherlands
- Jacqueline van de Wetering, Department of Nephrology, Erasmus MC University Medical Centre, 3015 GD, Rotterdam, the Netherlands
- Martine CM Willems, Department of Surgery, Academic Medical Centre Amsterdam, 1105 AZ, Amsterdam, The Netherlands
- Arjan D van Zuilen, Department of Nephrology, Utrecht University Medical Centre, 3584 CX, Utrecht, The Netherlands
- Frank J. M. F. Dor, Department of Surgery, Erasmus MC University Medical Centre, 3015 GD, Rotterdam, The Netherlands; Department of Surgery, Academic Medical Centre Amsterdam, 1105 AZ, Amsterdam, The Netherlands
